# Bis(2-amino-3-carb­oxy­pyridinium) sulfate trihydrate

**DOI:** 10.1107/S1600536811010191

**Published:** 2011-03-23

**Authors:** Fadila Berrah, Amira Ouakkaf, Sofiane Bouacida, Thierry Roisnel

**Affiliations:** a Laboratoire de Chimie Appliquée et Technologie des Matériaux LCATM, Université Larbi Ben M’Hidi, 04000 Oum El Bouaghi, Algeria; b Unité de Recherche de Chimie de l’Environnement et Moléculaire Structurale, CHEMS, Faculté des Sciences Exactes, Université Mentouri Constantine 25000, Algeria; c Centre de Difractométrie X, UMR 6226 CNRS Unité Sciences Chimiques de Rennes, Université de Rennes I, 263 Avenue du Général Leclerc, 35042 Rennes, France

## Abstract

In the title compound, 2C_6_H_7_N_2_O_2_^+^·SO_4_^2−^·3H_2_O, there are two independent cations which are connected into N—H⋯O hydrogen-bonded dimers. In the crystal, O—H⋯O hydrogen-bonded sulfate–water sheets run parallel to (001) and are linked into a three-dimensional network *via* inter­molecular N—H⋯O and O—H⋯O hydrogen bonds through the 2-amino­nicotinium dimers. Further stabilization is provided by weak inter­molecular C—H⋯O hydrogen bonds. *R*_4_^3^(10) and *R*_2_^2^(8) graph-set rings are observed. The crystal studied was an inversion twin with refined components of 0.45 (6) and 0.55 (6).

## Related literature

For related compounds, see: Athimoolam & Rajaram (2005 ▶); Berrah *et al.* (2005, ▶ 2011*a* ▶,*b* ▶); Dobson & Gerkin (1997 ▶); Giantsidis & Turnbull (2000 ▶); Pawlukojc *et al.* (2007 ▶). For hydrogen-bond motifs, see: Bernstein *et al.* (1995 ▶); Etter *et al.* (1990 ▶). For background to hydrogen bonding, see: Desiraju (2003 ▶).
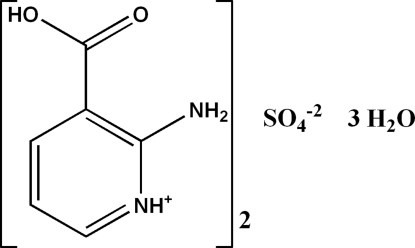


## Experimental

### 

#### Crystal data

2C_6_H_7_N_2_O_2_^+^·SO_4_^2−^·3H_2_O
*M* *_r_* = 428.39
Orthorhombic, 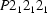

*a* = 6.5372 (5) Å
*b* = 12.3141 (10) Å
*c* = 23.0274 (19) Å
*V* = 1853.7 (3) Å^3^
*Z* = 4
Mo *K*α radiation
μ = 0.24 mm^−1^
*T* = 150 K
0.58 × 0.13 × 0.04 mm

#### Data collection

Bruker APEXII diffractometer
Absorption correction: multi-scan (*SADABS*; Sheldrick, 2002 ▶) *T*_min_ = 0.845, *T*_max_ = 0.970
23588 measured reflections
4229 independent reflections
3669 reflections with *I* > 2σ(*I*)
*R*_int_ = 0.042

#### Refinement

*R*[*F* ^2^ > 2σ(*F* ^2^)] = 0.031
*wR*(*F* ^2^) = 0.079
*S* = 1.06
4229 reflections
274 parameters
H atoms treated by a mixture of independent and constrained refinement
Δρ_max_ = 0.27 e Å^−3^
Δρ_min_ = −0.28 e Å^−3^
Absolute structure: Flack (1983 ▶), 1790 Friedel pairs
Flack parameter: 0.45 (6)

### 

Data collection: *APEX2* (Bruker, 2001 ▶); cell refinement: *SAINT* (Bruker, 2001 ▶); data reduction: *SAINT*; program(s) used to solve structure: *SIR2002* (Burla *et al.*, 2003 ▶); program(s) used to refine structure: *SHELXL97* (Sheldrick, 2008 ▶); molecular graphics: *ORTEP-3 for Windows* (Farrugia, 1997 ▶) and *DIAMOND* (Brandenburg & Berndt, 2001 ▶); software used to prepare material for publication: *WinGX* (Farrugia, 1999 ▶).

## Supplementary Material

Crystal structure: contains datablocks global, I. DOI: 10.1107/S1600536811010191/lh5219sup1.cif

Structure factors: contains datablocks I. DOI: 10.1107/S1600536811010191/lh5219Isup2.hkl

Additional supplementary materials:  crystallographic information; 3D view; checkCIF report

## Figures and Tables

**Table 1:** Hydrogen-bond geometry (Å, °)

*D*—H⋯*A*	*D*—H	H⋯*A*	*D*⋯*A*	*D*—H⋯*A*
O1*A*—H1*A*⋯O3*W*^i^	0.84	1.69	2.5152 (18)	167
O1*B*—H1*B*⋯O1*W*	0.84	1.69	2.5138 (18)	168
O1*W*—H1*W*⋯O2*W*^ii^	0.82 (4)	1.93 (3)	2.754 (2)	177 (4)
O3*W*—H5*W*⋯O2*W*	0.77 (3)	1.98 (3)	2.750 (2)	176 (3)
O1*W*—H2*W*⋯O4	0.75 (4)	2.03 (4)	2.752 (2)	164 (3)
O2*W*—H3*W*⋯O3^iii^	0.80 (3)	1.92 (3)	2.7151 (19)	169 (3)
O2*W*—H4*W*⋯O4	0.90 (3)	1.87 (3)	2.7675 (19)	175 (3)
O3*W*—H6*W*⋯O2^iv^	0.84 (2)	1.88 (2)	2.720 (2)	171 (3)
N2*A*—H2*A*⋯O1	0.88	1.92	2.7681 (18)	163
N2*B*—H2*B*⋯O1^v^	0.88	1.88	2.7419 (19)	167
N1*A*—H11*A*⋯O4	0.88	2.05	2.915 (2)	166
N1*B*—H11*B*⋯O2^v^	0.88	1.94	2.817 (2)	173
N1*A*—H12*A*⋯O2*A*	0.88	2.09	2.726 (2)	129
N1*A*—H12*A*⋯O2*B*	0.88	2.27	2.979 (2)	138
N1*B*—H12*B*⋯O2*A*	0.88	2.25	2.963 (2)	138
N1*B*—H12*B*⋯O2*B*	0.88	2.10	2.733 (2)	128
C4*A*—H4*A*⋯O3^vi^	0.95	2.46	3.143 (2)	129
C4*B*—H4*B*⋯O3^vii^	0.95	2.31	3.169 (2)	150

Symmetry codes: (i) 
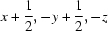
; (ii) 
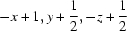
; (iii) 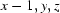; (iv) 
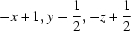
; (v) 
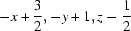
; (vi) 
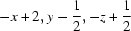
; (vii) 
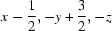
.

**Fig. 1.**
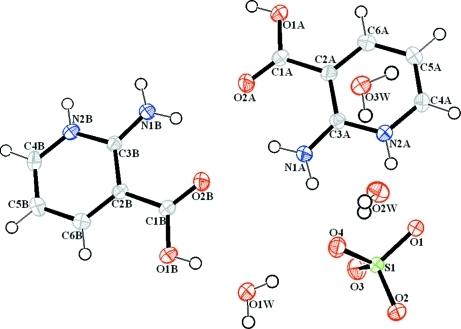
(Farrugia, 1997) The asymmetric unit of the title compound. Displacement are drawn at the 50% probability level.

**Fig. 2.**
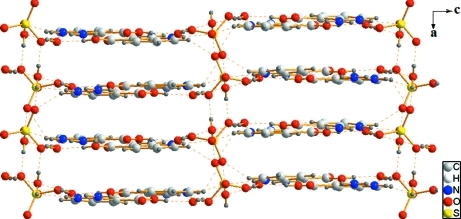
(Brandenburg & Berndt, 2001) A diagram of the three-dimentonal packing of (I) viewed along [010]. Hydrogen bonds are shown as dashed lines

**Fig. 3.**
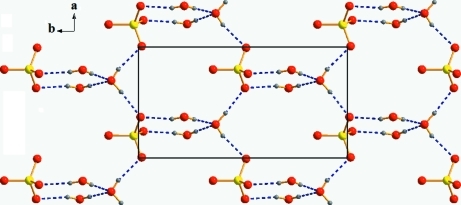
(Brandenburg & Berndt, 2001) A view of one sulfate-water sheet parallel to (001) and the R34(10) rings. Hydrogen bonds are shown as dashed lines.

**Fig. 4.**
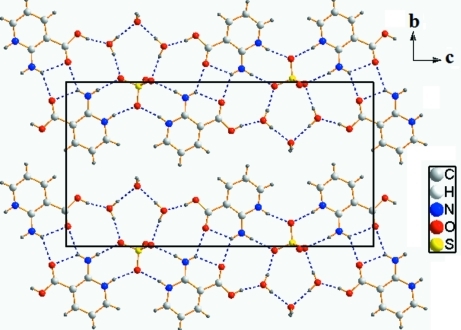
(Brandenburg & Berndt, 2001) Part of crystal packing showing cation dimers and R34(10) and R22(8) rings. Hydrogen bonds are shown as dashed lines.

## supplementary crystallographic information

### Comment

Hydrogen bonds are the object of several studies, which aim to elucidate their influence on crystal construction and compounds propreties (Desiraju, 2003). Pyridine and its derivatives well known for their various chemical and biological activities, have proved their aptitude to built new edifices involving original hydrogen-bonding patterns due to their variety of potential hydrogen donors and acceptors (Athimoolam *et al.*, 2005; Dobson & Gerkin, 1997; Giantsidis & Turnbull, 2000). The title compound was obtained from 2-aminonicotinic acid and is a part of our search for new hybrid compounds based on protonated N-heterocyclic compounds and inorganic acids (Berrah *et al.*, 2005;2011*a*,*b*).

As shown in figure 1, the asymmetric unit includes two crystallographically independent 2-aminonicotinium cations (A and B), one sulfate anion and three water molecules. The cation geometry is similar to that reported for the structure of 2-aminonicotinic acid (Dobson & Gerkin, 1997; Pawlukojc *et al.*, 2007) except for C—O distances in the carboxylic group. In the 2-aminonicotinic acid structure, the two C—O distances are 1.234 (2) and 1.266 (2)Å since the carboxylic group transfers its proton to the hetero-ring nitrogen atom leading to a zwitterionic molecule.

The crystal packing of the title compound (Fig. 2) results from sulfate-water sheets extending parallel to (001) (Fig. 3) and linked together *via* 2-aminonicotinium dimers (Fig. 4). In one sheet, sulfate anions and H_2_O2W molecules alternate, leading to infinite chains running parallel to the *a* axis. These chains are further connected through H_2_O1W and H_2_O3W molecules in a way that *R*^3^_4_(10) rings are formed (Fig. 3). The structure is stabilized *via* N—H···O, O—H···O and C—H···O Hydrogen bonds that link each dimer to its neighbors (Table 1, Fig. 4). *R*^3^_4_ (10) and *R*^2^_2_(8) graph-set rings are observed (Fig. 4)(Etter *et al.*, 1990; Bernstein *et al.*, 1995).

### Experimental

Colorless crystal of the title compound was obtained by slow evaporation of an aqueous solution of 2-amino-pyridine-3-carboxylic acid and sulfuric acid in 2:1 stoichiometric ratio.

### Refinement

The H atoms of the water molecules were located in difference Fourier maps and were refined with *U*_iso_(H) = 1.5*U*_eq_(O). The remaining H atoms were located in differnce Fourier maps but introduced in calculated positions and treated as riding on their parent atoms (C, N or O) with C—H = 0.95 Å, O—H = 0.84 Å and N—H = 0.88 Å with *U*_iso_(H) = 1.2 *U*_eq_(C or N) and *U*_iso_(H) = 1.5 *U*_eq_(O).

### Crystal data

**Table:**

2C_6_H_7_N_2_O_2_^+^·SO_4_^2^^−^·3H_2_O	*D*_x_ = 1.535 Mg m^−^^3^
*M**_r_* = 428.39	Mo *K*α radiation, λ = 0.71073 Å
Orthorhombic, *P*2_1_2_1_2_1_	Cell parameters from 8735 reflections
*a* = 6.5372 (5) Å	θ = 2.4–27.2°
*b* = 12.3141 (10) Å	µ = 0.24 mm^−^^1^
*c* = 23.0274 (19) Å	*T* = 150 K
*V* = 1853.7 (3) Å^3^	Needle, colourless
*Z* = 4	0.58 × 0.13 × 0.04 mm
*F*(000) = 896	

### Data collection

**Table:**

Bruker APEXII diffractometer	3669 reflections with *I* > 2σ(*I*)
graphite	*R*_int_ = 0.042
CCD rotation images, thin slices scans	θ_max_ = 27.5°, θ_min_ = 3.1°
Absorption correction: multi-scan (*SADABS*; Sheldrick, 2002)	*h* = −8→5
*T*_min_ = 0.845, *T*_max_ = 0.970	*k* = −15→15
23588 measured reflections	*l* = −29→29
4229 independent reflections	

### Refinement

**Table:**

Refinement on *F*^2^	Secondary atom site location: difference Fourier map
Least-squares matrix: full	Hydrogen site location: inferred from neighbouring sites
*R*[*F*^2^ > 2σ(*F*^2^)] = 0.031	H atoms treated by a mixture of independent and constrained refinement
*wR*(*F*^2^) = 0.079	*w* = 1/[σ^2^(*F*_o_^2^) + (0.0369*P*)^2^ + 0.4264*P*] where *P* = (*F*_o_^2^ + 2*F*_c_^2^)/3
*S* = 1.06	(Δ/σ)_max_ = 0.001
4229 reflections	Δρ_max_ = 0.27 e Å^−^^3^
274 parameters	Δρ_min_ = −0.28 e Å^−^^3^
0 restraints	Absolute structure: Flack (1983), 1790 Friedel pairs
Primary atom site location: structure-invariant direct methods	Flack parameter: 0.45 (6)

### Special details

**Table:**

Geometry. All e.s.d.'s (except the e.s.d. in the dihedral angle between two l.s. planes) are estimated using the full covariance matrix. The cell e.s.d.'s are taken into account individually in the estimation of e.s.d.'s in distances, angles and torsion angles; correlations between e.s.d.'s in cell parameters are only used when they are defined by crystal symmetry. An approximate (isotropic) treatment of cell e.s.d.'s is used for estimating e.s.d.'s involving l.s. planes.
Refinement. Refinement of *F*^2^ against ALL reflections. The weighted *R*-factor *wR* and goodness of fit *S* are based on *F*^2^, conventional *R*-factors *R* are based on *F*, with *F* set to zero for negative *F*^2^. The threshold expression of *F*^2^ > σ(*F*^2^) is used only for calculating *R*-factors(gt) *etc*. and is not relevant to the choice of reflections for refinement. *R*-factors based on *F*^2^ are statistically about twice as large as those based on *F*, and *R*- factors based on ALL data will be even larger.

### Fractional atomic coordinates and isotropic or equivalent isotropic displacement parameters (Å^2^)

**Table:**

	*x*	*y*	*z*	*U*_iso_*/*U*_eq_	
C1A	0.7166 (3)	0.27959 (15)	0.00318 (8)	0.0216 (4)	
C1B	0.6471 (3)	0.70977 (15)	0.03679 (8)	0.0200 (4)	
C2A	0.7181 (3)	0.23143 (14)	0.06245 (7)	0.0207 (4)	
C2B	0.6478 (3)	0.75822 (15)	−0.02254 (7)	0.0190 (4)	
C3A	0.7116 (3)	0.29935 (14)	0.11251 (7)	0.0201 (3)	
C3B	0.6631 (3)	0.68958 (14)	−0.07242 (7)	0.0190 (4)	
C4A	0.7238 (3)	0.14018 (15)	0.17195 (8)	0.0263 (4)	
H4A	0.726	0.1102	0.21	0.032*	
C4B	0.6481 (3)	0.84839 (16)	−0.13233 (8)	0.0262 (4)	
H4B	0.6479	0.8782	−0.1704	0.031*	
C5A	0.7264 (3)	0.07309 (16)	0.12519 (8)	0.0312 (5)	
H5A	0.728	−0.0036	0.1297	0.037*	
C5B	0.6322 (3)	0.91558 (16)	−0.08559 (8)	0.0282 (4)	
H5B	0.6211	0.992	−0.0904	0.034*	
C6A	0.7268 (3)	0.12083 (16)	0.07029 (9)	0.0279 (4)	
H6A	0.7333	0.0752	0.0371	0.034*	
C6B	0.6326 (3)	0.86880 (16)	−0.03039 (8)	0.0247 (4)	
H6B	0.6222	0.9145	0.0027	0.03*	
N1A	0.7020 (3)	0.40640 (12)	0.11129 (7)	0.0278 (3)	
H11A	0.7	0.4434	0.144	0.033*	
H12A	0.6976	0.4407	0.0778	0.033*	
N1B	0.6788 (3)	0.58296 (12)	−0.07142 (7)	0.0270 (4)	
H11B	0.6894	0.5465	−0.1041	0.032*	
H12B	0.6785	0.5482	−0.038	0.032*	
N2A	0.7181 (2)	0.24906 (12)	0.16507 (6)	0.0215 (3)	
H2A	0.7187	0.2899	0.1964	0.026*	
N2B	0.6642 (2)	0.73990 (13)	−0.12524 (6)	0.0225 (3)	
H2B	0.6761	0.6992	−0.1565	0.027*	
O1	0.7971 (2)	0.35697 (10)	0.26811 (5)	0.0259 (3)	
O1A	0.7192 (2)	0.20503 (10)	−0.03781 (5)	0.0294 (3)	
H1A	0.714	0.2352	−0.0705	0.044*	
O1B	0.6533 (2)	0.78383 (10)	0.07796 (5)	0.0277 (3)	
H1B	0.6398	0.7538	0.1105	0.042*	
O2	0.7597 (2)	0.52317 (11)	0.32163 (5)	0.0311 (3)	
O1W	0.6295 (3)	0.71875 (14)	0.18121 (6)	0.0467 (5)	
H1W	0.652 (5)	0.765 (3)	0.2061 (14)	0.07*	
H2W	0.653 (5)	0.664 (3)	0.1933 (14)	0.07*	
O2A	0.7137 (2)	0.37665 (11)	−0.00595 (5)	0.0296 (3)	
O2B	0.6414 (2)	0.61242 (10)	0.04572 (5)	0.0266 (3)	
O3	0.9930 (2)	0.51502 (12)	0.24034 (6)	0.0301 (3)	
O2W	0.3026 (2)	0.36878 (11)	0.23220 (6)	0.0270 (3)	
H3W	0.201 (4)	0.405 (2)	0.2347 (11)	0.04*	
H4W	0.405 (4)	0.417 (2)	0.2307 (11)	0.04*	
O4	0.6310 (2)	0.50965 (11)	0.22342 (5)	0.0259 (3)	
O3W	0.2063 (3)	0.23235 (12)	0.14171 (6)	0.0325 (3)	
H5W	0.239 (4)	0.271 (2)	0.1664 (11)	0.049*	
H6W	0.227 (4)	0.170 (2)	0.1557 (11)	0.049*	
S1	0.79804 (7)	0.47766 (4)	0.263808 (18)	0.01992 (10)	

### Atomic displacement parameters (Å^2^)

**Table:**

	*U*^11^	*U*^22^	*U*^33^	*U*^12^	*U*^13^	*U*^23^
C1A	0.0196 (8)	0.0255 (10)	0.0197 (8)	−0.0003 (8)	−0.0009 (7)	−0.0008 (7)
C1B	0.0188 (9)	0.0236 (10)	0.0176 (8)	0.0009 (7)	−0.0004 (7)	−0.0013 (7)
C2A	0.0205 (9)	0.0210 (9)	0.0207 (8)	−0.0023 (7)	−0.0018 (7)	−0.0009 (7)
C2B	0.0192 (9)	0.0212 (9)	0.0165 (8)	−0.0012 (7)	−0.0016 (7)	−0.0003 (7)
C3A	0.0203 (8)	0.0203 (8)	0.0196 (8)	−0.0009 (8)	−0.0001 (8)	0.0007 (7)
C3B	0.0205 (9)	0.0199 (9)	0.0167 (8)	0.0008 (7)	−0.0006 (7)	0.0007 (7)
C4A	0.0318 (10)	0.0239 (9)	0.0232 (9)	0.0002 (8)	−0.0036 (8)	0.0062 (8)
C4B	0.0300 (11)	0.0261 (10)	0.0225 (9)	0.0020 (8)	−0.0008 (8)	0.0081 (8)
C5A	0.0451 (12)	0.0192 (9)	0.0293 (10)	−0.0003 (9)	−0.0034 (9)	0.0025 (8)
C5B	0.0336 (11)	0.0214 (10)	0.0294 (10)	0.0018 (8)	−0.0012 (8)	0.0047 (8)
C6A	0.0354 (11)	0.0218 (9)	0.0266 (10)	0.0000 (8)	−0.0017 (9)	−0.0036 (8)
C6B	0.0278 (10)	0.0221 (9)	0.0241 (9)	0.0003 (8)	−0.0007 (8)	−0.0021 (8)
N1A	0.0465 (9)	0.0181 (8)	0.0188 (7)	0.0007 (8)	−0.0003 (8)	−0.0004 (6)
N1B	0.0436 (10)	0.0204 (8)	0.0171 (7)	0.0013 (8)	−0.0001 (7)	−0.0006 (6)
N2A	0.0256 (8)	0.0214 (7)	0.0175 (7)	0.0004 (7)	−0.0014 (6)	0.0000 (6)
N2B	0.0270 (8)	0.0243 (8)	0.0162 (7)	0.0004 (6)	0.0004 (6)	−0.0011 (6)
O1	0.0404 (7)	0.0191 (6)	0.0181 (6)	−0.0016 (6)	−0.0021 (6)	0.0004 (5)
O1A	0.0454 (8)	0.0252 (7)	0.0176 (6)	0.0011 (7)	−0.0006 (6)	−0.0015 (5)
O1B	0.0414 (8)	0.0250 (7)	0.0166 (6)	0.0008 (6)	−0.0005 (6)	−0.0018 (5)
O2	0.0508 (9)	0.0240 (7)	0.0184 (6)	0.0046 (6)	0.0021 (6)	−0.0021 (6)
O1W	0.0970 (15)	0.0245 (8)	0.0185 (7)	0.0102 (9)	−0.0078 (8)	−0.0008 (6)
O2A	0.0453 (8)	0.0226 (7)	0.0208 (6)	0.0020 (6)	0.0017 (6)	0.0026 (5)
O2B	0.0363 (8)	0.0224 (7)	0.0212 (7)	0.0017 (6)	0.0004 (6)	0.0031 (6)
O3	0.0274 (7)	0.0295 (8)	0.0332 (8)	−0.0038 (6)	0.0025 (6)	0.0054 (7)
O2W	0.0264 (7)	0.0248 (7)	0.0297 (7)	−0.0016 (6)	0.0013 (7)	−0.0017 (6)
O4	0.0284 (7)	0.0260 (7)	0.0234 (7)	−0.0015 (6)	−0.0034 (5)	0.0041 (6)
O3W	0.0538 (9)	0.0244 (8)	0.0193 (7)	−0.0026 (7)	−0.0042 (7)	−0.0014 (6)
S1	0.0259 (2)	0.0184 (2)	0.01552 (19)	−0.00133 (19)	−0.00044 (18)	0.00021 (17)

### Geometric parameters (Å, °)

**Table:**

C1A—O2A	1.214 (2)	C5B—C6B	1.395 (3)
C1A—O1A	1.317 (2)	C5B—H5B	0.95
C1A—C2A	1.488 (2)	C6A—H6A	0.95
C1B—O2B	1.217 (2)	C6B—H6B	0.95
C1B—O1B	1.316 (2)	N1A—H11A	0.88
C1B—C2B	1.491 (2)	N1A—H12A	0.88
C2A—C6A	1.375 (3)	N1B—H11B	0.88
C2A—C3A	1.425 (2)	N1B—H12B	0.88
C2B—C6B	1.377 (3)	N2A—H2A	0.88
C2B—C3B	1.430 (2)	N2B—H2B	0.88
C3A—N1A	1.320 (2)	O1—S1	1.4895 (13)
C3A—N2A	1.360 (2)	O1A—H1A	0.84
C3B—N1B	1.317 (2)	O1B—H1B	0.84
C3B—N2B	1.365 (2)	O2—S1	1.4662 (13)
C4A—N2A	1.351 (2)	O1W—H1W	0.82 (3)
C4A—C5A	1.357 (3)	O1W—H2W	0.75 (3)
C4A—H4A	0.95	O3—S1	1.4591 (14)
C4B—N2B	1.350 (2)	O2W—H3W	0.80 (3)
C4B—C5B	1.362 (3)	O2W—H4W	0.89 (3)
C4B—H4B	0.95	O4—S1	1.4877 (13)
C5A—C6A	1.394 (3)	O3W—H5W	0.77 (3)
C5A—H5A	0.95	O3W—H6W	0.85 (3)
			
O2A—C1A—O1A	124.23 (16)	C2A—C6A—C5A	122.46 (18)
O2A—C1A—C2A	123.47 (16)	C2A—C6A—H6A	118.8
O1A—C1A—C2A	112.30 (15)	C5A—C6A—H6A	118.8
O2B—C1B—O1B	124.18 (16)	C2B—C6B—C5B	121.86 (18)
O2B—C1B—C2B	123.32 (16)	C2B—C6B—H6B	119.1
O1B—C1B—C2B	112.51 (15)	C5B—C6B—H6B	119.1
C6A—C2A—C3A	118.44 (16)	C3A—N1A—H11A	120
C6A—C2A—C1A	121.03 (16)	C3A—N1A—H12A	120
C3A—C2A—C1A	120.52 (15)	H11A—N1A—H12A	120
C6B—C2B—C3B	118.95 (16)	C3B—N1B—H11B	120
C6B—C2B—C1B	121.05 (16)	C3B—N1B—H12B	120
C3B—C2B—C1B	120.00 (15)	H11B—N1B—H12B	120
N1A—C3A—N2A	118.38 (15)	C4A—N2A—C3A	123.86 (15)
N1A—C3A—C2A	124.77 (15)	C4A—N2A—H2A	118.1
N2A—C3A—C2A	116.85 (15)	C3A—N2A—H2A	118.1
N1B—C3B—N2B	117.89 (16)	C4B—N2B—C3B	123.82 (16)
N1B—C3B—C2B	125.49 (15)	C4B—N2B—H2B	118.1
N2B—C3B—C2B	116.61 (15)	C3B—N2B—H2B	118.1
N2A—C4A—C5A	120.77 (17)	C1A—O1A—H1A	109.5
N2A—C4A—H4A	119.6	C1B—O1B—H1B	109.5
C5A—C4A—H4A	119.6	H1W—O1W—H2W	109 (3)
N2B—C4B—C5B	120.77 (17)	H3W—O2W—H4W	105 (2)
N2B—C4B—H4B	119.6	H5W—O3W—H6W	104 (3)
C5B—C4B—H4B	119.6	O3—S1—O2	111.42 (9)
C4A—C5A—C6A	117.56 (18)	O3—S1—O4	109.05 (8)
C4A—C5A—H5A	121.2	O2—S1—O4	109.93 (8)
C6A—C5A—H5A	121.2	O3—S1—O1	110.06 (9)
C4B—C5B—C6B	117.98 (18)	O2—S1—O1	108.68 (7)
C4B—C5B—H5B	121	O4—S1—O1	107.62 (8)
C6B—C5B—H5B	121		
			
O2A—C1A—C2A—C6A	−178.3 (2)	C1B—C2B—C3B—N2B	−179.57 (16)
O1A—C1A—C2A—C6A	1.6 (3)	N2A—C4A—C5A—C6A	1.1 (3)
O2A—C1A—C2A—C3A	1.3 (3)	N2B—C4B—C5B—C6B	−0.1 (3)
O1A—C1A—C2A—C3A	−178.75 (18)	C3A—C2A—C6A—C5A	1.0 (3)
O2B—C1B—C2B—C6B	173.11 (18)	C1A—C2A—C6A—C5A	−179.38 (18)
O1B—C1B—C2B—C6B	−6.9 (2)	C4A—C5A—C6A—C2A	−2.1 (3)
O2B—C1B—C2B—C3B	−6.5 (3)	C3B—C2B—C6B—C5B	−0.1 (3)
O1B—C1B—C2B—C3B	173.45 (16)	C1B—C2B—C6B—C5B	−179.72 (17)
C6A—C2A—C3A—N1A	−179.65 (19)	C4B—C5B—C6B—C2B	−0.2 (3)
C1A—C2A—C3A—N1A	0.7 (3)	C5A—C4A—N2A—C3A	1.0 (3)
C6A—C2A—C3A—N2A	1.1 (3)	N1A—C3A—N2A—C4A	178.57 (18)
C1A—C2A—C3A—N2A	−178.53 (16)	C2A—C3A—N2A—C4A	−2.1 (3)
C6B—C2B—C3B—N1B	179.90 (18)	C5B—C4B—N2B—C3B	0.9 (3)
C1B—C2B—C3B—N1B	−0.5 (3)	N1B—C3B—N2B—C4B	179.60 (18)
C6B—C2B—C3B—N2B	0.8 (3)	C2B—C3B—N2B—C4B	−1.2 (3)

### Hydrogen-bond geometry (Å, °)

**Table:**

*D*—H···*A*	*D*—H	H···*A*	*D*···*A*	*D*—H···*A*
O1A—H1A···O3W^i^	0.84	1.69	2.5152 (18)	167
O1B—H1B···O1W	0.84	1.69	2.5138 (18)	168
O1W—H1W···O2W^ii^	0.82 (4)	1.93 (3)	2.754 (2)	177 (4)
O3W—H5W···O2W	0.77 (3)	1.98 (3)	2.750 (2)	176 (3)
O1W—H2W···O4	0.75 (4)	2.03 (4)	2.752 (2)	164 (3)
O2W—H3W···O3^iii^	0.80 (3)	1.92 (3)	2.7151 (19)	169 (3)
O2W—H4W···O4	0.90 (3)	1.87 (3)	2.7675 (19)	175 (3)
O3W—H6W···O2^iv^	0.84 (2)	1.88 (2)	2.720 (2)	171 (3)
N2A—H2A···O1	0.88	1.92	2.7681 (18)	163
N2B—H2B···O1^v^	0.88	1.88	2.7419 (19)	167
N1A—H11A···O4	0.88	2.05	2.915 (2)	166
N1B—H11B···O2^v^	0.88	1.94	2.817 (2)	173
N1A—H12A···O2A	0.88	2.09	2.726 (2)	129
N1A—H12A···O2B	0.88	2.27	2.979 (2)	138
N1B—H12B···O2A	0.88	2.25	2.963 (2)	138
N1B—H12B···O2B	0.88	2.10	2.733 (2)	128
C4A—H4A···O3^vi^	0.95	2.46	3.143 (2)	129
C4B—H4B···O3^vii^	0.95	2.31	3.169 (2)	150
C6A—H6A···O1A	0.95	2.35	2.697 (2)	101
C6B—H6B···O1B	0.95	2.37	2.709 (2)	100

Symmetry codes: (i) *x*+1/2, −*y*+1/2, −*z*; (ii) −*x*+1, *y*+1/2, −*z*+1/2; (iii) *x*−1, *y*, *z*; (iv) −*x*+1, *y*−1/2, −*z*+1/2; (v) −*x*+3/2, −*y*+1, *z*−1/2; (vi) −*x*+2, *y*−1/2, −*z*+1/2; (vii) *x*−1/2, −*y*+3/2, −*z*.
